# Targeting Melasma: Innovations in Pigment Deposition and Photoaging in Cosmetic Dermatology

**DOI:** 10.1111/jocd.70665

**Published:** 2026-01-08

**Authors:** Ting Liao, Rui Luo, Ying Deng, Hongqiu Yang, Yu Du

**Affiliations:** ^1^ Medical Cosmetic Center the Affiliated Traditional Chinese Medicine Hospital, Southwest Medical University Luzhou People's Republic of China; ^2^ Department of Acupuncture and Rehabilitation the Affiliated Traditional Chinese Medicine Hospital, Southwest Medical University Luzhou People's Republic of China

**Keywords:** hormonal influences, laser therapy, melasma, photoaging, pigmentation, topical treatments, UV exposure

## Abstract

**Background:**

Melasma is a chronic, relapsing hyperpigmentation disorder driven by complex interactions among genetic predisposition, hormonal fluctuations, UV exposure, oxidative stress, inflammation, and photoaging. Its psychosocial impact is substantial, especially among women with darker skin types, and treatment remains challenging due to its multifactorial pathogenesis and high recurrence rates.

**Aims:**

This review aims to synthesize current knowledge on melasma pathophysiology, highlight recent innovations in topical and procedural therapies, identify limitations of existing treatments, and outline future directions toward personalized and regenerative approaches.

**Patients/Methods:**

A narrative literature review was performed, summarizing findings from epidemiologic studies, molecular investigations, and clinical trials. Key topics included pigment biology, hormonal and environmental triggers, oxidative stress pathways, emerging depigmenting agents, laser and microneedling technologies, combination therapies, and advances in precision medicine.

**Results:**

Melasma arises from dysregulated melanogenesis involving hyperactive melanocytes, hormonal stimulation, UV‐induced oxidative stress, dermal inflammation, fibroblast senescence, and pigmentary incontinence. Innovations include non‐hydroquinone brighteners (e.g., thiamidol, melasyl), antioxidants, tranexamic acid, and improved topical delivery systems. Procedural advances—such as low‐fluence picosecond lasers, fractional CO_2_ laser–assisted drug delivery, chemical peel hybrids, and exosome‐augmented microneedling—offer enhanced pigment clearance with improved safety profiles. Nevertheless, treatment durability remains limited by relapse, heterogeneous disease subtypes, PIH risk, and inconsistent adherence.

**Conclusions:**

Melasma management is evolving toward multi‐target, combination‐based strategies addressing pigmentation, inflammation, and photoaging simultaneously. Emerging regenerative technologies, biomarker‐driven personalization, and AI‐assisted assessment hold promise for improving long‐term outcomes, though standardized protocols and long‐term safety data are still needed.

## 1 Introduction

### 1.1 Why Compose This Literature Review?

Melasma is one of the most common and widespread forms of hyperpigmentation, especially in women, and is predominantly seen in individuals with darker skin types (Fitzpatrick skin types III–VI) [1]. Although it is not a life‐threatening condition, its psychological and social impacts can be profound. Studies [2, 3, 4] consistently report that individuals with melasma experience a decrease in self‐esteem, confidence, and quality of life due to the visible and persistent nature of the pigmentary lesions, which primarily affect the face, often around the forehead, cheeks, and upper lip. Given its high prevalence, particularly among women of reproductive age, melasma poses a significant concern in dermatology [5, 6]. Melasma affects up to one‐third of women in sun‐exposed populations worldwide, with the highest prevalence reported in tropical regions [7, 8].

Despite various therapeutic strategies, melasma continues to be notoriously difficult to manage [9, 10, 11], and its chronicity often leads to frustration among patients and healthcare providers. The primary reason for this difficulty is the multifactorial nature of melasma's pathogenesis [10, 12, 13], which involves both intrinsic factors, such as genetic predisposition and hormonal influences, and extrinsic factors, such as UV exposure and photoaging. In recent years, significant progress [14, 15] has been made in our understanding of the underlying mechanisms of melasma, and this has led to the development of several innovative treatment approaches. However, the quest for an effective, long‐lasting, and safe solution remains an unmet need.

### 1.2 What Has Been Known?

Melasma is a common hyperpigmentation disorder that predominantly affects women with darker skin types (Fitzpatrick III–VI) and often leads to significant psychosocial distress due to its chronic, relapsing nature [1, 2, 3, 4]. Despite its benign course, melasma remains therapeutically challenging because of its multifactorial pathogenesis, which includes genetic, hormonal, and environmental influences such as ultraviolet (UV) exposure and photoaging [5, 6, 7, 8, 16].

Several treatment modalities are currently available for managing melasma, including topical agents [3] such as hydroquinone, retinoids, and corticosteroids. Chemical peels [17] and laser treatments [18, 19], such as fractional CO_2_ lasers and Q‐switched lasers, have also gained popularity in recent years. However, these treatments are not universally effective, and melasma often recurs after the cessation of therapy [20, 21]. Furthermore, the side effects [22, 23] associated with some treatments, such as skin irritation, ochronosis (a blue‐black discoloration of the skin caused by hydroquinone) [24], and increased pigmentation from lasers [18, 25], have prompted ongoing efforts to refine and develop more effective treatments.

### 1.3 What Has Not Been Known?

While substantial progress has been made in understanding the pathophysiology of melasma, significant gaps remain in our knowledge. For example, the precise molecular mechanisms by which hormonal fluctuations influence melanogenesis are not fully understood [14]. It is well‐established that estrogen and progesterone can increase the production of melanin in melanocytes [26], but the underlying signaling pathways remain an area of active investigation. Similarly, although oxidative stress and inflammatory responses induced by UV radiation are known to play a significant role in melasma, the exact pathways that link photoaging with melanocyte activation and pigment deposition are still not completely elucidated.

Additionally, while genetic studies have identified some key genes involved in melanin synthesis and pigmentation, the full spectrum of genetic factors contributing to melasma has not been comprehensively mapped. The role of epigenetics [27, 28], how environmental factors such as UV exposure may modify genetic expression, also remains an area of exploration.

On the therapeutic front, while numerous treatment options are available, there is still no universally effective therapy for melasma, and relapse rates remain high [29]. Current treatments [30], including topical agents like hydroquinone, often have side effects, and laser therapies can be ineffective [31, 32] or exacerbate pigmentation [11, 18] in certain skin types. Moreover, there is a lack of consensus on the most effective treatment regimen [33, 34], particularly for patients with darker skin types who are more prone to post‐inflammatory hyperpigmentation.

### 1.4 Literature Gap and Rationale for Writing

While progress has been made in understanding melasma, important gaps remain regarding the molecular links between hormonal influence, oxidative stress, and aging. This review synthesizes recent findings on the molecular mechanisms of melasma and emerging therapeutic innovations, with emphasis on novel topical agents, procedural advances, and regenerative medicine approaches.

Given the substantial impact of melasma on patients' quality of life and the increasing demand for effective treatments, this review will also discuss the need for more personalized treatment approaches that take into account the unique genetic, environmental, and clinical factors of each patient. By consolidating current knowledge, identifying gaps in existing treatments, and discussing future directions, this review will contribute to a deeper understanding of melasma and inform clinical practice in dermatology. Subsequent sections explore both classical and emerging treatments, emphasizing innovations that address melasma's multifactorial pathogenesis.

## 2 Pathophysiology of Melasma

### 2.1 Overview of Melasma Pathogenesis

Melasma is a complex skin disorder characterized by the overproduction and deposition of melanin, leading to hyperpigmented patches on the skin, primarily on sun‐exposed areas of the face [14]. Its pathogenesis is multifactorial, involving genetic, hormonal, and environmental factors that contribute to the dysregulation of melanogenesis [35]. At the core of melasma lies melanocytes [36], specialized skin cells responsible for the synthesis of melanin, the pigment that gives skin its color. Under normal circumstances, melanocytes respond to UV radiation by increasing melanin production as a protective mechanism. However, in melasma, melanocytes become hyperactive [37], producing excessive melanin even in the absence of excessive UV exposure. Melasma arises from an interplay of intrinsic (genetic and hormonal) and extrinsic (UV and environmental) factors that dysregulate melanogenesis [14].

### 2.2 Genetic Factors in Melasma

Genetics plays a significant role in the development of melasma, with family history being a strong indicator of risk. Epidemiological studies [38, 39] have shown that individuals with a family history of melasma are more likely to develop the condition. A twin study [40] has also demonstrated a hereditary component, suggesting that genetic predisposition may increase susceptibility to melasma. Several studies [41, 42] have identified potential genetic markers associated with skin pigmentation, including variations in genes such as MC1R (melanocortin 1 receptor) [43, 44], which is involved in the regulation of melanin synthesis.

However, while genetics is a key factor, it does not solely account for the development of melasma. It is thought that the combination of genetic predisposition and environmental factors [45], particularly UV exposure, triggers the onset of the condition. The interaction between genetic markers and external stimuli such as UV radiation has been suggested as a critical pathway in melasma development.

Moreover, epigenetic mechanisms [28]—changes in gene expression that do not involve alterations to the underlying DNA sequence—may also play a role in melasma. Epigenetic modifications [28] induced by environmental exposures such as UV light and pollution may lead to the increased expression of genes involved in melanogenesis, even in genetically predisposed individuals. This area of research is still in its early stages but holds promise for understanding the complex mechanisms driving melasma.

### 2.3 Hormonal Influences on Melanin Production

Hormonal factors are considered one of the most significant contributors to melasma. Hormonal fluctuations [14], especially those that occur during pregnancy, oral contraceptive use, or hormone replacement therapy, are commonly associated with the onset or exacerbation of melasma. The condition is often referred to as “chloasma” or “the mask of pregnancy” because of its frequent occurrence during pregnancy. Estrogen and progesterone have been shown to play a critical role in melasma pathogenesis [26]. These hormones influence melanocyte activity and promote the production of melanin, especially in individuals who are genetically predisposed to the condition.

Estrogen and progesterone receptors are present in melanocytes, and studies have demonstrated that elevated levels of these hormones can activate melanogenesis pathways [26]. Specifically, estrogen increases the activity of tyrosinase, the enzyme responsible for the initial steps in melanin production [46], while progesterone seems to enhance melanocyte proliferation. These hormonal effects are most evident during pregnancy, but oral contraceptives and hormone replacement therapy can also induce melasma in susceptible individuals.

Interestingly, sun exposure in conjunction with hormonal changes appears to amplify the risk of melasma [13]. UV radiation triggers an inflammatory response that activates melanocytes, and when this occurs alongside elevated estrogen and progesterone levels, the resulting effect is an exaggerated melanin response, leading to the formation of hyperpigmented patches.

### 2.4 UV Exposure, Photoaging, and Intrinsic Skin Aging in Melasma

UV radiation is one of the most well‐established environmental triggers of melasma. UV exposure leads to the formation of reactive oxygen species (ROS), which cause oxidative stress in skin cells [47]. This oxidative stress activates signaling pathways that stimulate melanogenesis by increasing the expression of tyrosinase and other enzymes involved in melanin synthesis [41]. The relationship between UV radiation and melanin production is well‐documented: the skin produces melanin as a protective response to UV radiation, with the goal of absorbing UV light and preventing DNA damage.

However, in melasma, this process becomes dysregulated. Excessive or chronic UV exposure can cause melanocytes to become hyperactive, producing excessive amounts of melanin. This leads to the characteristic pigmentary patches seen in melasma, particularly on the face [48, 49]. Interestingly, individuals with darker skin types are at higher risk for developing melasma because their melanocytes are more likely to respond aggressively to UV radiation, producing more melanin.

Moreover, photoaging—the premature aging of the skin due to UV exposure—plays a critical role in melasma. Photoaging results in the degradation of collagen and elastin fibers in the dermis, leading to a breakdown of the skin's structural integrity [50]. This breakdown creates a more favorable environment for melanocytes to proliferate and produce melanin. UV‐induced photoaging also leads to the formation of wrinkles, fine lines, and pigmentation changes, which further exacerbate melasma.

Recent studies [35, 51] have explored the role of UV‐induced inflammation in melasma. UV radiation not only activates melanocytes but also triggers an inflammatory cascade that contributes to the chronicity of melasma. Inflammatory mediators such as interleukins, prostaglandins, and tumor necrosis factor‐alpha (TNF‐α) have been implicated in this process [52]. This inflammatory response increases melanocyte activity, creating a feedback loop that perpetuates pigmentation and the progression of melasma.

Emerging evidence indicates that melasma is not solely a consequence of photoaging but also reflects features of intrinsic, chronological skin aging. Recent studies have demonstrated that senescent dermal fibroblasts release paracrine mediators—such as stem‐cell factor, endothelin‐1, and hepatocyte growth factor—that enhance melanogenesis in neighboring melanocytes. In addition, extracellular‐matrix remodeling and reduced collagen I/III turnover create a dermal microenvironment that favors pigment retention. Oxidative stress acts as a unifying mechanism between intrinsic and photo‐induced aging, amplifying reactive oxygen species–mediated signaling and chronic low‐grade inflammation. Collectively, these findings support the concept of melasma as an aging‐related pigmentary disorder rather than merely a photodamage response.

### 2.5 The Role of Melanophages and Pigmentary Incontinence

Building upon the aging‐related dermal alterations described above. In melasma, dysregulated epidermal melanogenesis leads to the overproduction of melanin, some of which subsequently deposits within the dermis. Importantly, melasma does not contain melanophages containing melanin derived from epidermal melanocytes. Instead, the pigment found in the dermis results from melanin produced by epidermal melanocytes that has leaked through a compromised basement membrane and been engulfed by dermal macrophages, known as melanophages. This process, termed pigmentary incontinence, explains the dermal component of melasma pigmentation and contributes to its chronic and treatment‐resistant nature [35, 53]. This form of pigmentary deposition is referred to as pigmentary incontinence [53], where melanin is transferred from damaged or dysfunctional melanocytes in the epidermis to the dermis.

Pigmentary incontinence is therefore a key feature of melasma with a dermal component. It occurs when melanin produced in the epidermis descends into the dermis because of inflammation‐induced disruption of the basement membrane. Dermal macrophages then phagocytose this melanin, forming melanophages that store pigment deep in the skin, making melasma harder to treat [53].

### 2.6 Inflammation and Oxidative Stress in Melasma

Oxidative stress and inflammation are central to the pathogenesis of melasma. UV‐induced ROS not only affect melanocytes but also lead to the production of pro‐inflammatory cytokines and chemokines that promote the inflammatory process in the skin. Studies [54, 55] have shown that oxidative stress increases the expression of melanin‐synthesizing enzymes, such as tyrosinase, thereby increasing melanin production. Additionally, the production of matrix metalloproteinases (MMPs) [56], which degrade the extracellular matrix, is elevated in response to UV‐induced oxidative stress, further promoting inflammation and contributing to photoaging.

The inflammatory process in melasma can be chronic, leading to the persistent nature of the condition. The overexpression of pro‐inflammatory cytokines such as IL‐1β and TNF‐α exacerbates melanocyte activation [57], perpetuating the cycle of hyperpigmentation. Inflammatory mediators have also been shown to disrupt the normal skin barrier, which may further contribute to the increased penetration of UV radiation and oxidative stress, worsening the condition.

## 3 Innovations in Melasma Treatment

### 3.1 Topical Therapies

Topical treatments remain the cornerstone of melasma management. These therapies aim to reduce melanin production, decrease pigmentation, and inhibit the inflammatory processes that exacerbate the condition. The most commonly used topical agents for melasma include hydroquinone, retinoids, corticosteroids, and more recently, tranexamic acid and nicotinamide. Below, we explore each of these treatments in detail.

#### 3.1.1 Hydroquinone

Hydroquinone remains the gold standard depigmenting agent because it inhibits tyrosinase and melanin synthesis [58, 59, 60]. However, long‐term use can cause irritation and exogenous ochronosis, so it is now employed in short courses or combined regimens rather than as monotherapy [61, 62, 63].

#### 3.1.2 Retinoids

Topical retinoids such as tretinoin enhance epidermal turnover and improve penetration of other agents, thereby lightening superficial pigmentation [64]. Their irritation potential limits use in sensitive skin, but they remain a valuable adjunct when properly titrated [58, 65].

#### 3.1.3 Corticosteroids

Topical corticosteroids (e.g., fluocinolone acetonide) suppress inflammation and melanocyte activity, particularly in triple‐combination formulas. Because chronic use can cause atrophy or rebound pigmentation, short‐term or intermittent application is recommended [66, 67].

#### 3.1.4 Tranexamic Acid

Tranexamic acid, traditionally used as an antifibrinolytic agent to prevent excessive bleeding, has recently emerged as a promising treatment for melasma [68]. Tranexamic acid works by inhibiting the plasminogen‐mediated conversion of plasminogen to plasmin, which in turn reduces the release of inflammatory mediators and inhibits melanocyte activation [68]. Its mechanism of action is believed to be both anti‐inflammatory and antioxidant, making it an effective option for patients with melasma exacerbated by UV‐induced oxidative stress.

Tranexamic acid is available in both topical and oral formulations, with studies demonstrating that both forms are effective in reducing melasma pigmentation [68]. Topical tranexamic acid has the advantage of fewer systemic side effects, while oral tranexamic acid is generally reserved for more severe cases. Clinical trials have shown that tranexamic acid can significantly reduce pigmentation and is well tolerated by most patients, although long‐term studies on its safety and efficacy are still ongoing.

#### 3.1.5 Nicotinamide (Niacinamide)

Nicotinamide, a form of vitamin B3, has been shown to possess anti‐inflammatory, antioxidant, and skin‐brightening properties [69]. It inhibits the transfer of melanin from melanocytes to surrounding keratinocytes, reducing the appearance of hyperpigmentation. Recent clinical investigation [70] has demonstrated that topical nicotinamide can improve the overall tone of the skin, making it a promising adjunct to other treatments for melasma.

Nicotinamide is generally well tolerated and has a favorable side effect profile. It is particularly useful in cases of melasma where inflammation plays a significant role in pigmentation. Furthermore, because it does not cause skin irritation, it can be used long‐term without the risk of adverse reactions, making it a good option for patients with sensitive skin or those unable to tolerate stronger treatments like hydroquinone or retinoids [69]. In addition to these established agents, several newer topical compounds have emerged in the past few years with promising depigmenting efficacy.

#### 3.1.6 Emerging Depigmenting Agents: Vitamin C, Arbutin, Thiamidol, and Melasyl

Recent advances in topical therapy have expanded the options beyond traditional agents such as hydroquinone and retinoids. Vitamin C and its derivatives (ascorbic acid, magnesium ascorbyl phosphate, and ascorbyl glucoside) act as potent antioxidants that directly inhibit tyrosinase and neutralize reactive oxygen species generated by UV exposure, thereby reducing oxidative stress–induced melanogenesis [71, 72, 73]. Arbutin, a naturally occurring β‐d‐glucopyranoside of hydroquinone, competitively blocks tyrosinase and is widely used in cosmeceutical lightening products because of its favorable safety profile [5, 24]. Thiamidol (isobutylamido thiazolyl resorcinol) is a next‐generation tyrosinase inhibitor shown in multiple randomized clinical trials (2023–2025) to significantly reduce MASI scores with minimal irritation [3, 24]. Melasyl (4‐(1‐phenylethyl)‐1,3‐benzendiol), a recently introduced small‐molecule inhibitor of melanin polymerization, interferes with dopaquinone conversion and prevents pigment maturation. Early clinical studies (2024–2025) demonstrate encouraging lightening effects when combined with daily photoprotection [5, 59]. Collectively, these novel compounds complement or substitute hydroquinone in patients requiring long‐term therapy or maintenance regimens. Incorporating antioxidants and non‐hydroquinone brighteners aligns with current trends toward safer, multi‐mechanistic treatment of melasma.

### 3.2 Procedural Treatments

Beyond topical agents, several advanced procedural techniques have recently emerged, offering enhanced pigment clearance and dermal rejuvenation through laser‐based, chemical, and microneedling approaches. While topical therapies are commonly used, more invasive procedures are often considered for patients who do not respond well to topical treatments. These procedures can provide faster results, but they also come with increased risk of complications, especially in individuals with darker skin types. Below, we explore the most commonly used procedural treatments for melasma: laser therapy, chemical peels, and microneedling.

#### 3.2.1 Recent Advances in Laser Technology Have Expanded the Therapeutic Options for Melasma

Low‐fluence picosecond lasers (730 and 1064 nm) deliver ultrashort energy pulses that fragment melanin granules with minimal thermal diffusion, reducing the risk of post‐inflammatory hyperpigmentation (PIH) [74, 75, 76]. The 675 nm fractional laser has emerged as another promising tool, targeting both pigment and dermal remodeling while improving skin elasticity and photoaged texture [10, 11]. In addition, laser‐assisted drug delivery now allows topical agents such as tranexamic acid, vitamin C, or cysteamine to penetrate deeper via microchannels created by fractional lasers [49, 68]. A cutting‐edge approach involves fractional CO_2_ laser combined with exosome therapy, which enhances pigment clearance and accelerates dermal repair through exosome‐mediated regeneration pathways [77, 78, 79]. These innovations provide more precise, safer, and synergistic options when tailored to the patient's skin type and pigmentation depth.

#### 3.2.2 Chemical Peeling Techniques Have Evolved to Provide More Controlled and Safer Exfoliation for Melasma

Newer formulations combine lactic acid plus pyruvic acid or modified medium‐depth TCA peels, which allow uniform penetration with reduced irritation compared to traditional glycolic acid peels [17, 75]. These blends promote gentle keratolysis and stimulate dermal renewal, leading to gradual pigment reduction and improved skin tone. Sequential or combination peels (e.g., glycolic followed by salicylic‐mandelic) are increasingly favored to minimize downtime and PIH risk, especially in darker skin types. Careful patient selection and strict photoprotection remain essential to prevent post‐procedure rebound pigmentation.

#### 3.2.3 Microneedling

Microneedling (collagen‐induction therapy) has evolved into a versatile platform for enhancing transdermal delivery of depigmenting agents. Recent studies demonstrate that microneedling combined with tranexamic acid, platelet‐rich plasma (PRP), or topical cysteamine provides superior pigment lightening compared with microneedling alone [50, 76, 80]. The microchannels created by the needles facilitate uniform absorption of active compounds and stimulate collagen synthesis, improving both pigmentation and skin texture. Devices integrating radiofrequency or fractional energy delivery further boost efficacy by promoting dermal remodeling while minimizing epidermal injury. Nonetheless, strict post‐procedure sun protection and gentle aftercare are critical to avoid treatment‐induced hyperpigmentation.

While microneedling is generally well tolerated, it is not without risks. Patients with melasma should be cautious, as the treatment may exacerbate pigmentation in some cases, especially when done incorrectly or with improper aftercare. As with chemical peels and laser treatments, microneedling should be performed by skilled professionals to minimize the risk of complications.

### 3.3 Combination Therapy

Given the multifactorial nature of melasma, a combination approach often yields the best results [81]. This may include combining topical therapies with procedural treatments to address different aspects of the condition simultaneously. For example, a patient may undergo laser treatment to target dermal melanin and use hydroquinone or tranexamic acid topically to treat epidermal pigmentation. Combination therapies help to enhance the overall effectiveness of treatment while minimizing the risk of recurrence.

Clinical studies have shown that combination therapy is particularly effective for refractory melasma, which does not respond well to monotherapy [81]. By targeting both the pigment‐producing melanocytes and the inflammatory processes that exacerbate the condition, combination treatments can provide more durable and satisfactory results for patients.

## 4 Challenges and Limitations in Melasma Treatment

Despite the numerous treatment options available for melasma, significant challenges remain in achieving consistent and long‐lasting results. These challenges are rooted in the multifactorial nature of the condition, as well as the limitations of current therapies. Below, we discuss the major obstacles faced by clinicians and patients in managing melasma, including issues related to the pathophysiology, treatment efficacy, safety, and patient adherence [82, 83].

### 4.1 Variability in Melasma Severity and Subtypes

One of the primary challenges in treating melasma is the heterogeneity of the condition. Melasma can vary greatly in terms of severity, location, and depth of pigmentation, with different patients presenting distinct clinical subtypes [63]. Generally, melasma is categorized into epidermal, dermal, and mixed types, each with its own unique characteristics [84]. Epidermal melasma is typically more superficial and involves increased pigmentation in the epidermis (the outermost layer of skin). This type generally responds well to treatments like hydroquinone, chemical peels, and laser therapy. Dermal melasma involves deeper pigmentation in the dermis, often requiring more aggressive treatment strategies. However, dermal melasma is notoriously difficult to treat and tends to be more resistant to conventional therapies, as melanocytes in the dermis are less responsive to topical treatments. Mixed melasma is the most common form and combines both epidermal and dermal pigmentation [84]. The presence of both types makes this form of melasma particularly challenging to treat, as different treatment modalities are often required to target both the epidermis and dermis.

Because of the differences in severity and type, standardized treatment protocols are difficult to establish. What works for one patient may not be effective for another, and the lack of a one‐size‐fits‐all approach makes it challenging to determine the best treatment plan.

### 4.2 Risk of Recurrence

Even after successful treatment, melasma often recurs, particularly when treatment is discontinued. The chronic nature of the condition, combined with continued UV exposure, makes relapse rates high. Several factors [85] contribute to the recurrence of melasma, including: Hormonal fluctuations: Since melasma is often hormonally induced, the recurrence of melasma is common after pregnancy, discontinuation of oral contraceptives, or hormone replacement therapy. UV exposure: Patients who do not consistently adhere to sun protection protocols are at high risk for recurrence, as UV radiation is a key factor in the development and exacerbation of melasma. Discontinuation of treatment: Many patients discontinue treatments when visible improvement is achieved, but failure to maintain treatment can lead to a return of pigmentation. Topical agents like hydroquinone require long‐term use for sustained results, and patients may not adhere to this regimen.

Despite improvements in treatment options, long‐term management remains a challenge, as even effective therapies often do not provide permanent results [86]. This recurrence emphasizes the importance of maintenance therapy, which is often neglected by patients who seek quick fixes rather than long‐term solutions.

### 4.3 Limitations of Current Topical Therapies

While topical treatments like hydroquinone, retinoids, and tranexamic acid are effective for many patients, they come with significant limitations [62]. Hydroquinone: As discussed earlier, hydroquinone remains the most commonly used agent in melasma treatment, but its potential for side effects, such as ochronosis (especially with prolonged use), has led to concerns about its long‐term safety. In some countries, hydroquinone has been banned or restricted due to its adverse effects. Retinoids: While retinoids can help to improve skin texture and promote epidermal turnover, they can cause skin irritation, dryness, and increased sensitivity to sunlight. This can make their use problematic, particularly in patients who already suffer from sensitive or dry skin. Tranexamic acid: Though promising, tranexamic acid's mechanism of action is not fully understood, and the long‐term safety of its use, especially in oral form, remains unclear. The oral form, in particular, carries risks, such as the potential for thromboembolic events (e.g., deep vein thrombosis or pulmonary embolism), particularly in patients with underlying risk factors for clotting disorders.

Moreover, while topical agents can reduce the appearance of pigmentation, they do not address the underlying mechanisms that contribute to melasma, such as hormonal imbalances or UV‐induced skin damage [87]. This leaves many patients with only partial and temporary results, especially when melasma is severe or dermal in nature.

### 4.4 Risks Associated With Procedural Treatments

Procedural treatments like laser therapy, chemical peels, and microneedling offer the potential for faster and more significant results compared to topical therapies. However, these treatments come with inherent risks and complications, particularly for patients with darker skin types (Fitzpatrick skin types IV–VI) [58]. Laser therapy: While lasers such as Q‐switched and fractional CO_2_ lasers are effective at targeting melanin and reducing pigmentation, they carry the risk of post‐inflammatory hyperpigmentation (PIH), a common complication where the skin becomes darker after treatment. This is particularly problematic for patients with darker skin tones, who are more prone to developing PIH. Additionally, inappropriate laser settings can exacerbate melasma, making it more difficult to treat. Chemical peels: Although chemical peels can be effective in exfoliating the skin and reducing pigmentation, they can also lead to skin irritation, erythema (redness), and PIH, particularly in patients with sensitive or darker skin. The depth and strength of the peel must be carefully considered to avoid adverse effects, and patients must be closely monitored after treatment. Microneedling: Microneedling, when combined with PRP or topical agents, has shown promise in treating melasma by stimulating collagen production and improving skin texture. However, the procedure can exacerbate pigmentation in some cases, especially if performed incorrectly or without appropriate post‐treatment care [30]. Moreover, its effectiveness in treating dermal melasma is still being evaluated.

Although procedural treatments can provide faster results, they require skilled practitioners to minimize the risk of complications. Furthermore, the cost and accessibility of these treatments may limit their use, particularly for patients without the financial means or access to high‐quality care.

### 4.5 Patient Adherence and Lifestyle Factors

One of the most significant challenges in managing melasma is ensuring patient adherence to treatment regimens [88]. Many patients fail to follow through with long‐term treatment plans, either due to side effects, lack of immediate results, or the chronic nature of the condition. This is particularly true for topical agents like hydroquinone and retinoids, which require consistent, long‐term application.

Additionally, lifestyle factors such as sun exposure and hormonal fluctuations play a crucial role in melasma's progression and recurrence [14, 58, 78]. While patients are often advised to use sunscreen daily, many neglect this crucial step or fail to apply it correctly. Similarly, women who experience melasma during pregnancy or while using oral contraceptives may find that their condition returns once these hormonal factors come into play again.

Educating patients about the chronic nature of melasma and the importance of consistent sun protection, as well as maintenance therapy, is critical for improving treatment outcomes. However, non‐adherence remains a significant barrier, as many patients seek immediate solutions rather than long‐term management strategies.

### 4.6 Lack of Consensus on Treatment Protocols

Despite the wide variety of treatments available, there is no universal consensus on the optimal treatment protocol for melasma. While some dermatologists may recommend topical agents as first‐line therapy, others may prioritize laser treatments or chemical peels. The lack of standardized guidelines can lead to variability in treatment outcomes, as patients may receive different recommendations depending on their healthcare provider's expertise and preferences [33].

Furthermore, there is a need for more robust, long‐term clinical studies to establish clear treatment protocols and evaluate the comparative efficacy of different approaches. Most current studies focus on short‐term outcomes, leaving the long‐term effectiveness and safety of treatments largely unaddressed.

## 5 Future Directions in Melasma Treatment

The management of melasma is continuously evolving, with new treatment modalities, advancements in technology, and a deeper understanding of its pathophysiology driving future directions. The quest for more effective, targeted, and personalized treatment approaches is at the forefront of melasma research [89]. Below, we discuss some of the most exciting developments in the field, including novel treatment options, the role of genomics and biomarkers, artificial intelligence (AI) in treatment optimization, and the future of combination therapies.

### 5.1 Personalized and Precision Medicine

Regenerative and combination‐based therapies are redefining melasma management. Techniques incorporating stem‐cell‐derived exosomes, platelet‐rich plasma (PRP), and fractional CO_2_–assisted drug delivery promote dermal remodeling and pigment clearance through cellular rejuvenation [90, 91]. Similarly, AI‐assisted image analysis is improving lesion assessment and individualized treatment selection [92, 93, 94, 95]. These innovations highlight a paradigm shift toward biologically guided, precision interventions.

As our understanding of the genetic, hormonal, and environmental factors contributing to melasma deepens, the shift toward personalized medicine is gaining momentum. Personalized treatment approaches involve tailoring therapies based on an individual's unique genetic makeup, environmental exposures, and skin characteristics [58, 96]. Rather than relying on a “one‐size‐fits‐all” treatment approach, this methodology seeks to maximize treatment efficacy by accounting for the patient's specific disease pathogenesis.

Recent studies [41, 97] have shown that certain genetic markers, such as variations in the MC1R gene, may influence melanin synthesis and pigmentation patterns. This has prompted interest in using genetic testing to predict how an individual's skin will respond to various melasma treatments. By identifying patients at higher risk of developing melasma or those who may be more likely to respond to specific therapies, clinicians could make more informed treatment decisions, improving both efficacy and patient satisfaction.

Moreover, biomarkers that predict melasma severity or treatment response are an area of active research. The identification of such biomarkers could allow for better monitoring of treatment progress and potentially enable the development of targeted therapies. For example, markers of oxidative stress or inflammatory mediators could help assess the impact of UV exposure and inflammation on melasma progression, providing insights into the effectiveness of treatments like antioxidants or anti‐inflammatory agents.

### 5.2 Advancements in Laser and Light‐Based Therapies

Laser and light‐based treatments have become increasingly popular in the management of melasma due to their ability to target the underlying pigment directly [76]. However, the risk of complications, particularly post‐inflammatory hyperpigmentation (PIH) in darker skin types, remains a significant barrier. In response to these challenges, ongoing innovations in laser technology are aimed at improving both efficacy and safety.

One exciting development is the advent of fractional lasers, which deliver energy in a pixelated pattern, allowing for precise treatment of the skin while minimizing thermal damage. Fractional CO_2_ lasers and fractional erbium lasers have shown promise in treating both epidermal and dermal melasma by targeting melanin deep within the skin without causing excessive damage to surrounding tissue [75]. These lasers stimulate collagen production, which not only improves the appearance of melasma but also enhances overall skin texture and tone.

Additionally, pulsed light therapies, such as intense pulsed light (IPL), are being investigated for their ability to treat melasma with minimal risk of hyperpigmentation in darker skin types [98]. IPL works by targeting the pigment in the skin, selectively destroying melanin while leaving the surrounding tissue unharmed. Clinical trials have shown that IPL can effectively reduce pigmentation with fewer side effects than traditional lasers.

Researchers [99, 100] are also exploring the use of picosecond lasers, which emit extremely short bursts of light to break down melanin into smaller fragments that can be absorbed by the skin. Preliminary studies suggest that picosecond lasers may be more effective than traditional nanosecond lasers, with a reduced risk of PIH and better results for dermal melasma [101]. The integration of fractional CO_2_ lasers with exosome or tranexamic acid delivery illustrates how procedural and biologic innovations are converging to create next‐generation combination therapies for melasma.

### 5.3 Topical Innovations and New Drug Formulations

While existing topical treatments such as hydroquinone and retinoids have demonstrated efficacy, their limitations in terms of side effects and long‐term effectiveness have led to the exploration of new drug formulations and topical agents [30]. Recent research has focused on developing alternatives to hydroquinone that are not only effective but also have fewer adverse effects [102].

One promising agent is mequinol [103], a skin‐lightening compound that works similarly to hydroquinone but has a lower risk of causing ochronosis. Study had shown that mequinol, when combined with tretinoin [104], can provide effective results in the treatment of melasma, with a lower risk of side effects compared to hydroquinone.

Another exciting development is the use of vitamin C and its derivatives [72, 73], such as ascorbic acid and ascorbyl glucoside, in melasma treatment. Vitamin C is a powerful antioxidant that not only inhibits tyrosinase but also helps to neutralize free radicals generated by UV exposure, thereby reducing the oxidative stress that exacerbates melasma. Clinical studies [105, 106] have demonstrated the effectiveness of vitamin C in lightening pigmentation and improving overall skin radiance.

Topical tranexamic acid has also emerged as a promising treatment, particularly for patients with resistant melasma [107, 108]. Tranexamic acid inhibits the plasminogen pathway, reducing the inflammatory response and melanocyte activity triggered by UV exposure. Its topical form has gained traction as a less invasive alternative to oral tranexamic acid, providing similar results with fewer systemic side effects. New non‐hydroquinone depigmenting molecules such as thiamidol and melasyl further expand the therapeutic armamentarium for melasma and exemplify the shift toward safer, multi‐target approaches.

### 5.4 Artificial Intelligence (AI) in Melasma Diagnosis and Treatment

The application of artificial intelligence (AI) in dermatology has shown great potential in improving the diagnosis, monitoring, and treatment of melasma [92]. AI algorithms are being developed to assist in the early detection of melasma by analyzing images of the skin and identifying pigmentation patterns that may be indicative of the condition [93]. By using machine learning models, these algorithms can accurately assess melasma severity and provide personalized treatment recommendations based on the patient's unique characteristics.

Moreover, AI‐driven tools are being integrated into dermatological imaging systems, allowing for non‐invasive, high‐resolution imaging of the skin [94]. These systems can monitor changes in pigmentation over time, providing clinicians with real‐time feedback on treatment effectiveness. AI can also help predict the risk of recurrence by analyzing data on a patient's hormonal history, UV exposure, and genetic factors, leading to more proactive management [95].

Additionally, AI may play a role in optimizing treatment regimens by analyzing patient data and predicting the most effective combination of therapies for melasma, as other disorders [109, 110]. This could help reduce trial‐and‐error approaches in melasma treatment and improve outcomes for patients.

### 5.5 Regenerative Medicine and Stem Cell Therapy

Regenerative medicine is an emerging field that holds significant promise for the future treatment of melasma [90, 111]. A recent research exploring the use of stem cells and platelet‐rich plasma (PRP) shows that both stem cells and PRP are able to promote skin regeneration and reduce pigmentation [90]. The idea is that by stimulating collagen production and improving skin healing, regenerative therapies could not only reduce melasma pigmentation but also improve overall skin texture and resilience.

For example, adipose‐derived stem cells (stem cells derived from fat tissue) are being investigated for their potential to restore damaged skin and improve pigmentation [112]. Studies [113, 114] have shown that stem cell‐based treatments can promote wound healing and collagen synthesis, which may help in the treatment of both epidermal and dermal melasma.

Similarly, PRP therapy, which involves using a patient's own blood plasma enriched with platelets to promote tissue regeneration, is being explored for its ability to improve skin tone and reduce pigmentation [91]. When combined with microneedling, PRP can enhance the absorption of topical treatments and promote skin rejuvenation [115, 116].

### 5.6 Combination Therapies for Enhanced Efficacy

Given the complex, multifactorial nature of melasma, combination therapies are likely to become the standard of care in the future [117]. The integration of multiple treatment modalities can target different aspects of melasma's pathogenesis, leading to more effective and durable outcomes.

Combination therapies may involve the use of topical agents (such as hydroquinone, tranexamic acid, or retinoids) alongside procedural treatments (such as laser therapy or microneedling) to address both the epidermal and dermal components of melasma [80]. Furthermore, combining therapies that target inflammation, oxidative stress, and melanin synthesis could provide more comprehensive treatment options.

Recent studies have demonstrated that combining anti‐inflammatory agents (like corticosteroids or niacinamide) with melanin inhibitors (like hydroquinone or tranexamic acid) results in improved pigmentation control and reduced risk of recurrence. The combination of laser therapy with topical agents has also shown promising results [80], as it can enhance the penetration of therapeutic agents and promote collagen synthesis.

## 6 Conclusion

Melasma is a multifactorial and chronic dermatologic condition characterized by the overproduction and deposition of melanin, which results in hyperpigmented patches, primarily on sun‐exposed areas of the face. Although melasma is not a life‐threatening condition, it poses significant aesthetic concerns and profoundly affects the quality of life of those affected. The complex nature of melasma, driven by genetic, hormonal, and environmental factors, makes it a challenging condition to manage effectively. Despite the array of available treatment options, melasma remains a persistent and often recurrent condition, which underscores the need for continued advancements in its management.

This literature review has highlighted the key factors contributing to the pathogenesis of melasma, including genetic predisposition, hormonal influences, and UV exposure. Furthermore, the emerging role of photoaging in exacerbating melasma has been discussed, emphasizing the importance of addressing both pigmentation and the underlying skin damage caused by UV radiation. Understanding these complex interactions is crucial for developing more targeted and personalized treatment approaches.

Current treatment modalities, such as topical agents (e.g., hydroquinone, retinoids, tranexamic acid), laser therapies, and chemical peels, have shown varying degrees of success in treating melasma. However, each therapy comes with its own set of limitations, including side effects, relapse rates, and the risk of post‐inflammatory hyperpigmentation (PIH), particularly in patients with darker skin types. The challenge of achieving long‐lasting results, along with the difficulty of managing recurrent melasma, remains a significant concern in clinical practice.

Looking forward, the field of melasma treatment is poised for significant advancements. Personalized medicine offers the potential to tailor treatments based on individual genetic, hormonal, and environmental factors, improving both efficacy and patient satisfaction. Additionally, innovative approaches in laser therapy, topical treatments, and regenerative medicine hold promise for more effective and less invasive solutions. The integration of artificial intelligence (AI) in both the diagnosis and treatment planning of melasma could revolutionize how clinicians approach this condition, allowing for more precise and personalized interventions.

Combination therapies that target multiple aspects of melasma's pathogenesis—such as pigmentation, inflammation, and photoaging—are likely to become the standard of care, offering more durable and comprehensive results.

Furthermore, the identification of biomarkers and the development of novel drug formulations are essential for advancing the field and overcoming the limitations of current treatments.

Despite these promising developments, several challenges remain. The heterogeneity of melasma, the high recurrence rate, and the lack of standardized treatment protocols highlight the need for further research and clinical trials to establish effective, long‐term treatment regimens. Additionally, increasing patient awareness and education about the chronic nature of melasma, the importance of sun protection, and adherence to long‐term therapies is critical to improving treatment outcomes.

In conclusion, while melasma continues to be a difficult condition to treat, the future of its management looks promising. The integration of personalized treatment plans, the refinement of existing therapies, and the development of new, cutting‐edge treatments provide hope for more effective and sustainable solutions. As research continues to progress, it is likely that the treatment landscape for melasma will evolve, offering patients better outcomes and an improved quality of life.

## Author Contributions

Ting Liao and Rui Luo contributed equally to the conceptualization, literature review, and drafting of the manuscript. Ying Deng and Hongqiu Yang assisted with data collection, critical review of the literature, and refinement of the manuscript. Yu Du supervised the project, provided critical revisions for important intellectual content, and approved the final version of the manuscript.

## Funding

The authors have nothing to report.

## Ethics Statement

The authors have nothing to report.

## Consent

The authors have nothing to report.

## Conflicts of Interest

The authors declare no conflicts of interest.

## Data Availability

Data sharing not applicable to this article as no datasets were generated or analyzed during the current study.
